# Sleep Duration and Quality among Different Occupations--China National Study

**DOI:** 10.1371/journal.pone.0117700

**Published:** 2015-03-17

**Authors:** Wenjie Sun, Yaqin Yu, Jingqin Yuan, Changwei Li, Tingting Liu, Dongdong Lin, Abby Lau, Chongke Zhong, Tan Xu, GuangLiang Shan

**Affiliations:** 1 School of Food Science, Guangdong Pharmaceutical University, Zhongshan 528458, China; 2 School of Public Health and Tropical Medicine, Tulane University, New Orleans, LA, 70112, United States of America; 3 Department of Epidemiology, School of Public Health, Jilin University, Changchun, 130021, China; 4 Ningxia Autonomous Region Department of Public Health. Yinchuan, Ningxia, 750001, China; 5 Department of Epidemiology, Tulane University School of Public Health and Tropical Medicine. New Orleans, LA, 70112, United States of America; 6 Nell Hodgson Woodruff School of Nursing, Emory University, Atlanta, GA, United States of America; 7 School of Science and Engineering, Tulane University, New Orleans, LA, 70112, United States of America; 8 Department of Infectious Diseases, School of Medicine, Tulane University, New Orleans, LA, 70112, United States of America; 9 Department of Epidemiology, School of Public Health, Medical College of Soochow University, Suzhou, Jiangsu, 215123, China; 10 Department of Epidemiology and Statistics, Institute of Basic Medical Sciences, Chinese Academy of Medical Sciences; School of Basic Medicine, Peking Union Medical College, Beijing, 100005, China; Hospital General Dr. Manuel Gea González, MEXICO

## Abstract

**Objective:**

To examine the associations between occupation, sleep duration and sleep quality.

**Methods:**

The data for this study was extracted from data collected from the 2008 Chinese Sub-optimal Health Study. Our study sample consisted of 18,316 Chinese subjects aged 18-65. Occupation and other relevant characteristics to sleep were collected. We used the Pittsburgh Sleep Quality Index (PSQI) to measure sleep quality and multiple logistic regression models to examine the association of occupation with shortened sleep duration and poor sleep quality.

**Results:**

Farmers had the longest sleep duration (mean=8.22 hours) while the civil servants had the shortest sleep duration (mean=7.85 hours). Farmers also had the best sleep quality (mean score=3.74) while professional workers had the worst sleep quality (mean score=4.87). Compared to civil servants, the OR of shortened sleep duration and poor sleep quality for blue collar workers is 1.39 (95%CI: 1.11-1.73) and 1.28 (95%-CI: 1.15-1.42), respectively, after adjusting for age, sex, marital status, education, area, smoking, drinking, pain, and health status.

**Conclusion:**

sleep duration and quality varied among different Chinese occupation populations. The blue collar workers are more likely to have shortened sleep duration and poor sleep quality.

## Introduction

Decreased sleep duration has been shown to be associated with increased morbidity and mortality [1–3]. Research suggests that factors such as socio-demographic and comorbid health factors [4], psychosocial stress [5], and lifestyle [6] are associated with sleep quality and duration. Hence, sleep duration and quality are not only medicine issue but also a socio-demographic issue. For example, self-reported sleep duration varies by industry and occupation, according to the USA national health interview survey [7]. Furthermore, sleep duration varies between countries, races and ethnicities [8–11]. For example, according to data from the National Health Interview Survey from 2004–2001, Asians were more likely to report decreased sleep duration compared with Caucasians (33% vs. 28%)[12]. Sleep quality also has been found to vary by socioeconomic status (SES) and improves with increasing socioeconomic status [13]. Thus, white-collar workers reported the highest quality of sleep, compared with blue-collar workers who had the lowest quality of sleep[14]. However, thus far, no studies have investigated the association between occupation and sleep quality and duration.

We conducted a national study comparing sleep performance in Chinese workers in different occupations. We tested three hypotheses: 1) Sleep duration varies amongst different occupations; 2) Sleep quality varies amongst different occupations; 3) Occupation is an independent determinant of sleep duration and quality.

## Methods

### Participants

The China Sub-optimal Health Survey (CSHS) was created in 2008 to understand the changing health status of China based on a sample population. The CSHS selected individuals from 6 provinces to represent the 1.4 billion individuals in the nation's population. A multi-stage, random cluster sampling design was used to designate study subjects. All 31 provinces or municipalities were divided into 6 administrative regions (Northeast, North, East, Central South, Southwest, and Northwest). The regions of Jilin, Beijing, Jiangsu, Hubei, Sichuan and Gansu were randomly selected to represent those six administrative regions. Each of the above randomly selected regions was divided into multiple urban and suburban regions. Then, 1–2 urban regions and 1–2 suburban regions were randomly selected to represent both the urban and suburban populations. Within those selected regions, residents including local college students, government staff, business and farm workers and other non-affiliated local residents were clustered and randomly selected as the sample population.

19,665 participants were selected to participate in the study, of whom 18,631 responded and filled out questionnaires (response rate of 94.7%). We excluded individuals who 1) were less than 18 or greater than 65 years of age; 2) have mental illnesses that could potentially affect sleep patterns; and 3) were employed as shift workers. 18,284 participants were included in the final analysis.

### Ethics Statement

This study was approved by the Institutional Review Board at Peking Union Medical College and followed the tenets of the Declaration of Helsinki. Written informed consent was obtained from all participants.

### Data collection

All individuals in each randomly cluster selected unit were asked to completed a self-administered questionnaire. The data on demographic and personal characteristics were collected, including gender, age, marital status, education, smoking, drinking, and health information (medical history, illness and diseases that occurred during the last 12 months). Information on occupation was based on Chinese labor law.

### Sleep

#### Sleep duration

The number of hours of sleep was assessed by the following questions inserted into a self-reported questionnaire: “On average, when you go to bed at night?” and “On average, when you arise in the morning?”.

Sleep duration was calculated according to following formulas: Preferred sleep duration = (preferred arising time + 24) − (preferred going to bed time); Short sleep duration was defined as less than 6 hours, according to the sleep duration. The classification was consistent with the previous studies [15–17].

#### Sleep quality

The Pittsburgh Sleep Quality Index (PSQI) was used to estimate the participating’s sleep quality. The cutoff point of PSQI score was 5 (PSQI >5 indicate poor sleep quality) which was consistent with the previous studies [18,19].

### Statistic

Statistical analysis was carried out with Windows Statistical Software Package Version 10.0 (SAS Institute, Cary, NC, USA). Occupations were analyzed as a categorical variable. Sleep duration and sleep quality were categorized into two groups (cutoff points were 6 hours and 5 scores, respectively), which were investigated as binary outcome variables. Chi-square tests were used to compare participants’ characteristics by sleep duration and sleep quality. We used ANOVA (Analysis of variance) to test the first hypothesis that sleep duration or sleep quality would be varied by occupation. Tukey test was used to compare difference between the groups. Logistic regression model was applied to estimate the odds ratio and 95% CIs of short sleep duration and poor sleep quality by occupations adjusted for potential confounders. Potential confounders considered were sex, age, education, area, marriage, smoking, drinking, body pain, and health status, categorized as in Table 1 (A significance level of 0.05 is required to allow the variable enter the model). Health status was assessed based on self-reports of chronic illness including hypertension, diabetes, coronary heart disease, hyperlipidemia, hepatitis, and other diseases. Participants with any of the above chronic disease were labeled as "unhealthy". Two sets of potential confounders were used in the adjusted models. Model 1 adjusted for sex and age. Model 2 additional adjusted for education, area, marriage, smoking, drinking, body pain, and health status. All potential confounders were summarized in Table 1. All the tests were two sided and significance level was set at 0.05.

**Table 1:** Participant characteristics and frequencies (in %) within each sleep duration and quality category.

	Sleep duration (hours)			Sleep quality (score)		
	≤6	>6	Short duration prevalence	≤5	>5	Poor quality prevalence
Sex						
Men	50.16	59.55	6.92	51.54	48.47	25.85
Women	49.84	40.45	4.84	48.46	51.53	28.27
Age (years)						
18–25	28.71	25.52	5.27	29.67	25.44	24.12
25-≤45	55.08	49.72	5.35	54.08	56.58	27.94
45-≤65	16.21	24.76	8.73	16.25	17.98	29.09
Occupation						
Civil	16.74	16.82	5.92	17.00	16.07	25.94
Professional	14.81	15.03	5.98	14.17	16.58	30.25
Worker	26.91	30.62	6.65	25.84	30.61	30.51
Famer	9.38	6.24	4.00	9.70	7.83	23.02
Business man/service	8.77	9.36	6.26	9.22	7.70	23.66
Students	15.86	14.27	5.34	16.35	14.17	24.31
Others	7.52	7.66	5.99	7.71	7.04	25.30
Education						
Liberate/primary school	25.76	22.97	5.29	25.68	25.38	26.81
High school	23.09	25.24	6.41	22.9	24.08	28.05
College	51.15	51.8	5.96	51.43	50.55	26.7
Area						
Jilin	17.29	17.77	6.05	16.48	19.57	30.56
Gansu	15.5	19.09	7.16	13.49	21.71	37.36
Sichuan	17.48	15.31	5.20	17.51	16.95	26.41
Jiangsu	17.25	5.39	1.92	19.4	8.86	14.47
Hubei	16.92	22.59	7.71	16.12	20.31	31.83
Beijing	15.55	19.85	7.40	16.99	12.61	21.56
Marriage						
Single	33.76	31.10	5.45	34.48	31.25	25.15
Married	63.94	64.37	5.93	63.56	65.05	27.5
Devoice/separate/Widow	2.30	4.54	10.98	1.96	3.71	41.19
Smoking						
No	76.79	65.5	5.07	76.86	74.13	26.34
yes	23.21	34.5	8.51	23.14	25.87	29.3
Drinking						
No	70.07	58.22	4.95	69.82	68.16	26.57
Yes	29.93	41.78	8.04	30.18	31.84	28.11
Body pain						
No	32.31	47.45	4.64	25.13	54.97	18.23
Yes	67.69	52.55	8.42	74.87	45.03	44.77
Healthy						
Yes	78.75	67.77	5.11	80.42	71.82	24.87
No	21.25	32.23	8.67	19.58	28.18	34.79

## Results

The study included a total of 18,316 Chinese adults from 6 provinces with a mean age of 33.1 (SD = 10.6). 50.73% were male and 49.27% were female. Overall, 5.93% of study participants reported a sleep duration of less than 6 hours (male: 6.92%; female: 4.84%) and 26.98% reported poor sleep quality (male: 25.85%; female: 28.87%). The percentage of individuals who reported a sleep duration less than 6 hours (considered a "short sleep duration") was varied based on occupation, 5.92% of civil servants, 5.98% professional workers, 6.65% of blue collar workers, 4% of farmers, and 6.26% of business workers, 5.34% of students and 5.99% of other individuals reported short sleep duration. The percentage of individuals who reported a poor sleep quality was varied based on occupation. Based on the cut-off points using the PSQI recommendations, 25.94% of civil servants, 20.25% of professionals, 30.51% of blue collar workers and 23.02% of farmers, 23.66% of business workers, 24.31% of students and 25.30% of other individuals reported poor sleep quality.

Table 2 shows the distribution of sleep duration and sleep scores in the study participants. Farmers had the longest sleep duration (mean = 8.22 hours) while civil servants had the shortest sleep duration (mean = 7.85 hours). Farmers also had the best sleep quality (mean score = 3.74) while professional workers had the worst sleep quality (mean score = 4.87).

**Table 2:** Distribution of sleep duration sleep quality scores by occupation.

	Sleep duration (hour)		Sleep quality (score)	
Occupation	Mean	SD	Mean	SD
Civil	7.85	1.02	4.57	2.37
Professional	7.87	1.08	4.87*	2.41
Worker	8.10*	1.17	4.75*	2.55
Famer	8.22*	1.05	3.74*	2.76
Business man/service	8.20*	1.21	4.31*	2.32
Students	7.88	1.06	4.43	2.13
Others	8.07*	1.14	4.46	2.37

*Compared with civil, P<0.05

Table 3 shows the results of simple and multiple logistic regression models used to elucidate the effects of different demographic variables on the association of short sleep duration and poor sleep quality.

**Table 3:** Multiple logistic regression models and the associations between occupations, sleep duration, and sleep quality.

	Civil	Professional	Worker	Farmer	Business	Students	Others
Short sleep duration							
Model 1	Ref.	1.08 (0.87–1.36)	1.22 (1.01–1.48)*	0.70 (0.52–0.93)*	1.17 (0.90–1.51)	1.10 (0.84–1.45)	1.09 (0.83–1.44)
Model 2	Ref.	1.18 (0.94–1.47)	1.39 (1.11–1.73)*	0.83 (0.59–1.16)	1.41 (1.07–1.85)*	1.25 (0.94–1.67)	1.35 (1.01–1.81)*
Poor sleep quality							
Model 1	Ref.	1.24 (1.11–1.40)*	1.28 (1.15–1.42)*	0.87 (0.75–0.99)*	0.93 (0.80–1.07)	1.08 (0.93–1.24)	0.97 (0.85–1.14)
Model 2	Ref.	1.32 (1.17–1.50)*	1.20 (1.06–1.36)*	0.90 (0.75–1.07)	1.15 (0.98–1.34)	1.02 (0.87–1.19)	1.07 (0.90–1.26)

Model 1 adjusted sex, and age; Model 2 adjusted sex, age, education, area, marriage, smoking, drinking, body pain, and health status.

*P<0.05

### Association between occupation and sleep duration

The crude OR for short sleep duration is 1.22 (95% CI 1.01–1.48) for blue collar workers and 0.70 (95% CI 0.52–0.93) for farmers compared with civil. Additional adjustments based on education, area, marriage, smoking, drinking, body pain, and health status in model 2 did not attenuate the effect of occupation for civil (OR = 1.39; 95% CI: 1.11–1.73), but there was an attenuation of the effect of occupation for farmers (OR = 0.83; 95% CI: 0.59–1.16). In model 2, the adjusted OR for shortened sleep duration is 1.41 (95% CI: 1.07–1.85) for business compared with civil.

### Association between occupation and sleep quality

Similarly, the OR for poor sleep quality was 1.24 (95% CI: 1.11–1.40) for professional, and 1.28 (95% CI: 1.15–1.42) for blue collar workers, and 0.87 (95% CI: 0.75–0.99) for famers compared to that of civil. Additional adjustments based on education, area, marriage, smoking, drinking, body pain, and health status in model 2 did not attenuate the association amongst professional (OR = 1.32; 95% CI: 1.17–1.50) and blue collar workers (OR = 1.20; 95% CI: 1.06–1.36), but there was attenuation of this association found amongst farmers (OR = 0.90; 95% CI: 0.75–1.07).

## Discussion

In this nationally representative sample of the Chinese population, we found blue collar workers had a higher prevalence of shortened sleep duration and decreased sleep quality compared with that of individuals in other occupations. In multiple logistic regression analysis, blue collar workers and business workers had shorter sleep duration compared with individuals in other occupations. Professional workers and blue collar workers had decreased quality sleep compared with individuals in other occupations. Our study confirmed the results of a previous study which showed that shortened sleep duration varied based on industry and occupation among US workers [7].

There are a few potential explanations for these results. The "healthy worker" effect could explain why civil servants are healthier than blue collar workers. In China, all civil servants are required to pass a strict medical examination upon recruitment. Conversely, blue collar worker are only occasionally required to pass a medical examination, and these test requirements are lower compared to civil servants. Hence, civil workers could be healthier and they might have better sleep quality or long duration. However, in our analysis, we adjusted for health status and did not find that this changed the association found with occupation.

Another potential explanation for these results is based on health and sleep disparities due to differences in SES. Anders et al. conducted a cross-sectional study with 3,281 participants in Germany and found that low socio-economic status was associated with poor sleep quality They concluded that SES is one of the determinants of good sleep quality, but it is that not most important determinant and it does not act in isolation [13]. Of note, the SES in their study was calculated based on participants' education, net household income, and occupation. However, occupation was one of the important components in determining SES and highly associated with education level and household income. Occupation was more likely to be determined by education level and affect the household income. Hence, Anders et al. might have over-adjusted for the occupation independent effect of SES.

It is possible that occupation contributes to risk factors associated with shortened sleep duration and poor sleep quality, such as obesity[20], and other etiologies.

The strengths of this study include the large sample size and the adjustment for other important cofounders (education, health status, etc.). However, our study has several limitations. Sleep data was self-reported which can be imprecise and subject to reporting biases. This study did not include objective measurements of sleeps. Furthermore, there are no validated tools for assessing self-reported sleep duration. We lacked data on physical activity in subjects, which could be potential confounders. Finally, our study was performed on a relatively homogenous population. Further observational studies focusing on different populations using objective measures of sleep duration may help clarify this association between occupation and sleep.

In conclusion, our results suggest that Chinese workers have a short sleep duration and poor sleep quality compares with civil. occupations that contribute to differences in sleep duration and quality. These differences still need to be identified in the future study.
